# Combination of GT90001 and nivolumab in patients with advanced hepatocellular carcinoma: a multicenter, single-arm, phase 1b/2 study

**DOI:** 10.1186/s12916-023-03098-w

**Published:** 2023-10-20

**Authors:** Chiun Hsu, Yi-Fang Chang, Chia-Jui Yen, Yu-Wei Xu, Min Dong, You-Zhi Tong

**Affiliations:** 1 Department of Medical Oncology, National Taiwan University Cancer Center, No. 57, Ln. 155, Sec. 3, Keelung Road., Da’an Dist., Taipei, 106 Taiwan; 2 Department of Oncology, National Taiwan University Hospital, No. 7, Chung-Shan South Road, Taipei, 100 Taiwan; 3 Department of Hematology and Oncology, Mackay Memorial Hospital, Taipei, Taiwan; 4 Department of Oncology, National Cheng Kung University Hospital, College of Medicine, National Cheng Kung University, Taipei, Taiwan; 5 Suzhou Kintor Pharmaceuticals, Inc., Suzhou, China

**Keywords:** GT90001, PF-03446962, Nivolumab, Immunotherapy, Anti-angiogenic therapy, Hepatocellular carcinoma

## Abstract

**Background:**

GT90001 (also known as PF-03446962) is an anti-ALK-1 monoclonal antibody and has shown activity in hepatocellular carcinoma (HCC). This phase 1b/2 study was designed to determine the recommended phase 2 dose (RP2D) of GT90001 plus nivolumab, and assess the safety and anti-tumor activity in patients with advanced HCC.

**Methods:**

Patients with advanced HCC were recruited from 3 centers. Eligible patients in the dose de-escalation stage received the GT90001 on day 1 of a 14-day cycle in a rolling-six design with a fixed dose of nivolumab (3.0 mg/kg). Patients in dose-expansion stage received the RP2D of GT90001 plus nivolumab. Primary endpoint was safety. Key secondary endpoint was objective response rate (ORR) as per RECIST 1.1.

**Results:**

Between July 9, 2019, and August 8, 2022, 20 patients were treated (6 in phase 1b; 14 in phase 2) and evaluable for analysis. In phase 1b, no dose-limiting toxicities were observed, and GT90001 7.0 mg/kg was confirmed as the RP2D. Common grade 3/4 adverse events (AEs) were platelet count decreased (15%). No deaths due to AEs were reported. Confirmed ORR and disease control rate were 30% (95% CI, 14.6%-51.9%) and 40% (95% CI, 21.9%-61.3%), respectively. Median duration of response was not calculated (95% CI, 7.39 months to not calculated). Median progression-free survival (PFS) was 2.81 months (95% CI, 1.71–9.33), with 6-month and 12-month PFS rates of 35% and 25%, respectively. One patient with multiple intra- and extra-hepatic metastases was diagnosed with pseudo-progression upon GT90001 plus nivolumab exposure.

**Conclusions:**

GT90001 plus nivolumab has a manageable safety profile and promising anti-tumor activity in patients with advanced HCC.

**Trial registration number:**

ClinicalTrials.gov identifier NCT03893695.

**Supplementary Information:**

The online version contains supplementary material available at 10.1186/s12916-023-03098-w.

## Background

Immune checkpoint inhibitors (ICIs) targeting the programmed cell death-1 (PD1)/programmed cell death ligand-1 (PDL1) pathway constituted the backbone of systemic therapy for patients with un-resectable hepatocellular carcinoma (HCC) [1]. Many single-agent and combination regimens have been tested in the first-line and more advanced stage settings. Single-agent nivolumab or pembrolizumab produced an objective response rate of about 15% in patients with advanced HCC who had received sorafenib therapy [2, 3], and a randomized, placebo-controlled trial demonstrated survival benefit in Asian HCC patients who had progression on or intolerance to sorafenib or oxaliplatin-based chemotherapy [4]. The therapeutic benefit, in terms of both survival and objective response, may be further improved by combining anti-PD1/anti-PDL1 ICIs with anti-angiogenic targeted therapy [5–8]. Angiogenesis inhibition may exert immune modulatory effects in the tumor micro-environment through multiple mechanisms [9]. Therefore, ICIs plus anti-angiogenic agents are currently the most extensively tested immuno-oncology (IO)-based combination regimen for advanced HCC.

GT90001 (previously known as PF-03446962) is a monoclonal antibody targeting the activin receptor-like kinase 1 (ALK-1), a serine/threonine kinase receptor regulating angiogenesis through interaction with the transforming growth factor-β (TGFβ) signaling network [10]. Preclinical studies indicated that ALK-1 activity may contribute to resistance to inhibitors targeting the vascular endothelial growth factor receptor (VEGFR) signaling pathway, while anti-ALK-1 and anti-VEGFR may have antitumor synergy [11, 12]. In addition, TGFβ signaling may regulate antitumor immunity through multiple mechanisms. Increased TGFβ activity may suppress antitumor response through inhibiting CD4 + T helper cell function (T_H_1 and T_H_2), decreasing differentiation and function of cytotoxic T cells, promoting pro-tumor T_H_17 response, and recruiting immune suppressive myeloid cells into the tumor microenvironment [13]. The role of TGFβ in regulatory T cells, B cells, and innate immunity has also been extensively studied [13]. Combination of TGFβ inhibitors with anti-PD1 immune checkpoint inhibitors may produce synergistic antitumor activities in pre-clinical models [14, 15], providing rational for testing this combination in the clinic.

GT90001 has been evaluated in several early-phase clinical trials for patients with advanced solid tumors. The recommended dosage for phase 2 trials was 7 mg/kg intravenously every 2 weeks. The most common treatment-related adverse events demonstrated in these trials of GT90001 included thrombocytopenia, fatigue, and telangiectasia, which were also distinct from adverse associated with VEGFR inhibition [16–18]. Although single-agent antitumor activity in terms of objective response rate was absent, about 30% of patients with advanced HCC in these early-phase trials achieved stable disease lasting for more than 12 weeks, suggesting disease-stabilizing effects of GT90001 [18].

**Fig. 1:**
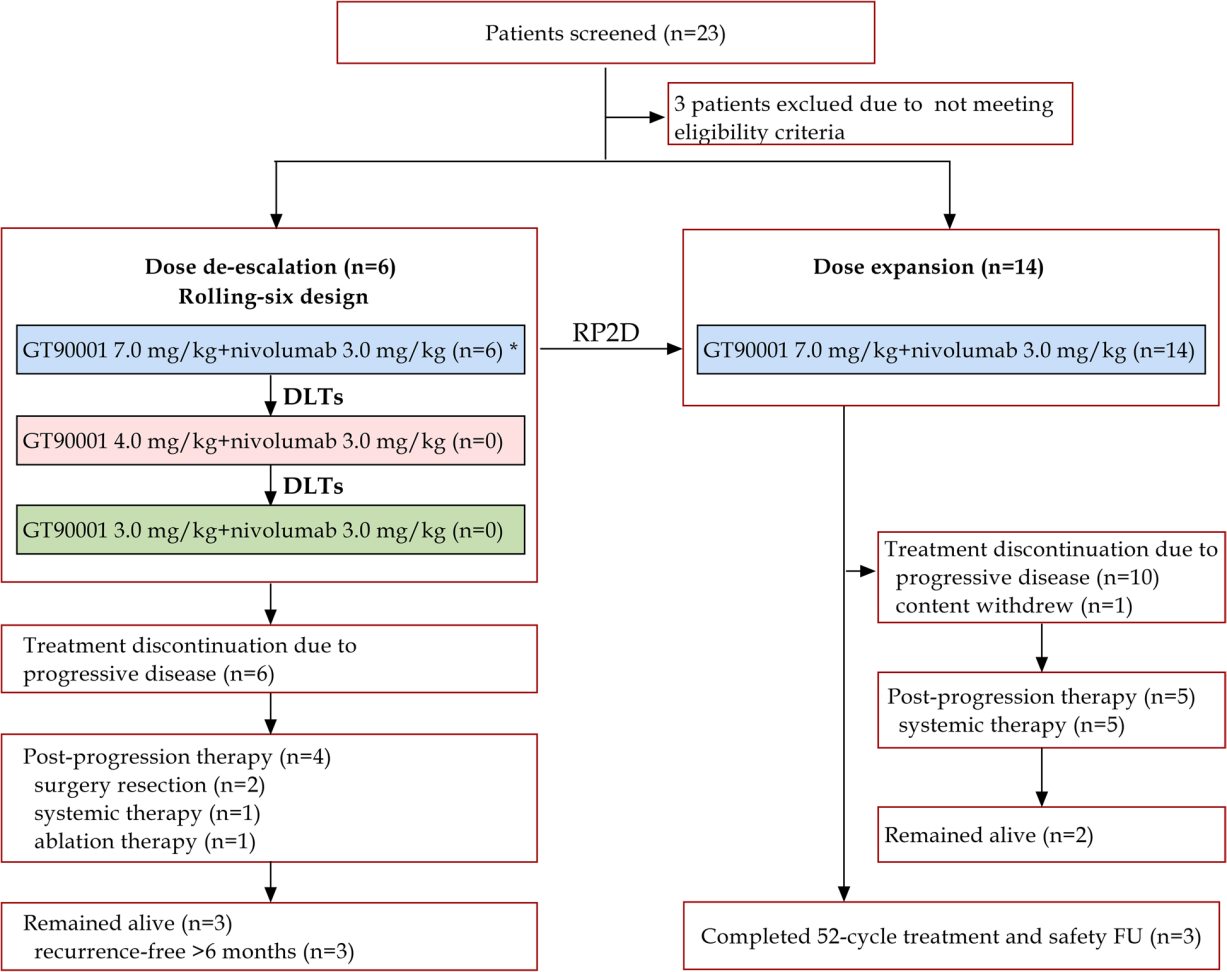
Study profile. *No DLTs were observed in the 7.0 mg/kg cohort. RP2D = recommended phase 2 dose; DLTs = dose-limiting toxicities

Based on the above evidence, we conducted a phase 1b/2 study of GT90001 combined with nivolumab to test the hypothesis that GT90001 may improve the therapeutic efficacy of anti-PD1 ICI therapy for patients with advanced HCC. This study consisted of a dose de-escalation stage to determine the recommended phase 2 dosage (RP2D) of GT-90001 in combination with the anti-PD1 nivolumab and a dose expansion stage to evaluate the safety and efficacy of GT-90001- nivolumab combination (Fig. 1).

## Methods

### Study design and participants

This single-arm, open-label trial for patients with unresectable HCC was conducted at 3 medical centers in Taiwan (ClinicalTrials.gov identifier NCT03893695). Key eligibility criteria included the following: age ≥ 20 years; histologically or cytologically confirmed unresectable HCC that was refractory or not amenable to locoregional therapy; disease progression or intolerance after first-line systemic treatment; at least one measurable tumor lesion according to Response Evaluation Criteria In Solid Tumors (RECIST) version 1.1 [19]; Child–Pugh score 5 or 6; Eastern Cooperative Oncology Group (ECOG) performance score 0 or 1; a life expectancy of at least 3 months; and adequate organ function. Key exclusion criteria included the following: liver tumors occupying > 50% of the liver volume; tumor invasion into bile ducts; tumor invasion or thrombosis at the main portal vein; and prior therapy with ICIs. Complete eligibility criteria are listed in the supplement (Appendix File 1: Table S1). This study was approved by the institutional review boards from all participating centers and was performed in accordance with the ethical principles that have their origin in the Declaration of Helsinki, and the International Council for Harmonisation Good Clinical Practice (ICH-GCP). All patients signed written informed consent prior to any study-related procedure.

### Procedures

Dose de-escalation phase. The primary objective of this phase was to evaluate the safety and the RP2D of GT90001 combined with nivolumab. A rolling-six design was used (Appendix File 1: Figure S1). Both GT90001 and nivolumab were given as a 60-min intravenous infusion (IV) on day 1 of a 14-day cycle. The dosage of nivolumab was fixed at 3.0 mg/kg. The initial dosage of GT90001 was 7.0 mg/kg, and 3 dose levels were planned (7.0, 4.5, and 3.0 mg/kg). Dose was only de-escalated when two or more dose-limiting toxicities (DLTs) were observed at a particular dose level. DLT was defined as any one of the following: (1) grade 4 platelet count decreased or grade 3 decrease with active bleeding; (2) grade 4 neutropenia, lymphopenia, anemia, alanine aminotransferase (ALT)/aspartate aminotransferase (AST) increased, alkaline phosphatase increased, vomiting, fatigue, rash, infection or fever (in the absence of neutropenia); (3) grade 3 nausea or vomiting lasting for > 3 days despite anti-emetic treatment; (4) grade 3 rash, infection or fever (in the absence of neutropenia) lasting for > 7 days; (5) life-threatening toxicity as determined by the principal investigator; (6) any other grade 3 or worse treatment-emergent adverse events (TEAEs) occurring within the first 28 days of treatment.

Dose-expansion phase. The primary objective of this phase was to assess the efficacy and safety of GT90001 at the recommended dosage in combination with nivolumab. After the RP2D was determined, additional patients were enrolled in the dose-expansion cohort (Appendix File 1: Figure S1) and treated at the RP2D for up to 2 years, until disease progression, the development of unacceptable toxicity, loss of clinical benefit, or withdrawal of consent.

Tumor response was evaluated by investigators according to RECIST 1.1. Assessments of tumor response were conducted by computed tomography (CT) or magnetic resonance imaging at baseline and scheduled for every 8 weeks within one year, then every 12 weeks thereafter (with a window of ± 1 week) until disease progression. Confirmation of partial response (PR) or complete response (CR) is required at 4 weeks after response, as per RECIST 1.1 [19].

Safety and tolerability were monitored throughout the trial and during the 30-day follow-up period (after the last administration of the study treatment). Adverse events (AEs) were graded according to the National Cancer Institute Common Terminology Criteria for Adverse Events (NCI-CTCAE 5.0) and incidence of any TEAEs, serious AEs (SAEs), treatment-related AEs, AEs leading to discontinuation, and deaths, were recorded. The following AEs were considered related to the mechanism of action of GT90001, including platelet count decreased, fatigue, fever, telangiectasia, amylase increased, lipase increased, epistaxis, nausea, chills, and headache. Patients were monitored by physical examination, chest radiography, ECOG performance score, vital signs, and laboratory analyses (hematology, serum biochemistry, urinalysis, and coagulation) at baseline, before cycles 1–3, every 8 weeks within one year, every 12 weeks thereafter, at the end of study treatment, and follow-up 30 days.

### Dose adjustments

If a patient experienced immune-related adverse events (irAEs) attributed to nivolumab, nivolumab was withheld or permanently discontinued depending on the types and severity of irAEs. Corticosteroids were administrated according to current guidelines. Dose reduction of nivolumab was not allowed in this study. The detailed dose modification regimen of GT90001 was listed as following: (1) no dose interruption or modification was made for grade 1 or 2 toxicity, regardless of non-hematologic or hematologic toxicity; (2) dose suspension or reduction is required or suggested for grade 3 non-hematologic non-DLTs (until toxicity returned to ≤ grade 1 or has returned to baseline) and grade 3 hematologic non-DLTs (until toxicity returned to ≤ grade 2 or has returned to baseline); (3) dose reduction or discontinuation is required or suggested for the following possibly-related toxicities: grade 3 non-hematologic DLTs or grade 4 non-hematologic toxicity (until the toxicity returned to ≤ grade 1 or has returned to baseline), and grade 3 hematologic DLTs or grade 4 hematologic toxicity (until the toxicity returned to ≤ grade 2 or has returned to baseline). If toxicity recovered to tolerable (grade 1 non-hematologic toxicities or grade 2 hematologic toxicities) within 4 weeks, protocol therapy could continue; if not, whether protocol therapy would be continued was judged by investigators.

### Outcomes and statistical analysis

The primary endpoint of this trial was AEs according to NCI-CTCAE 5.0. Secondary endpoints included objective response rate (ORR; CR and PR according to RECIST 1.1), the disease control rate (DCR; CR, PR, and stable disease [SD] ≥ 16 weeks according to RECIST 1.1), duration of response (DOR; the time from the first evidence of response until disease progression [PD] or death), time to response (TTR; the time from treatment initiation to the first evidence of verified response), and progression-free survival (PFS; time from treatment initiation to PD or death from any cause).

In the dose de-escalation phase, sample sizes for each dose were determined on the basis of the rolling-six design. In the dose-expansion phase, no formal hypothesis testing is pre-defined in this exploratory study. All patients in phase 1 and phase 2 studies were used for the analysis of primary and secondary endpoints. Patient characteristics, safety outcomes, and tumor response are summarized descriptively. Categorical variables are summarized by frequencies (percentage [%]) and continuous variables are summarized by medians (interquartile range [IQR]). Safety was assessed in all patients who received at least one dose of study medication and had at least one safety assessment. Efficacy analyses were assessed in the intention-to-treat population, defined as patients who received at least one dose of study medication. We estimated 95% confidence intervals (CIs) using the Clopper-Pearson method for tumor response. DOR, TTR, and PFS were analyzed by Kaplan–Meier methodology and expressed as medians with 95% CIs. All statistical tests were two-sided, significance set at *p* < 0.05. Statistical analyses were performed using SAS software (SAS Institute Inc, USA).

## Results

### Patient characteristics

Enrolment occurred from July 9, 2019 and June 26, 2020, and the data cutoff for analysis was August 8, 2022. A total of 23 patients with advanced HCC were screened and among them, 3 patients were ineligible (Child–Pugh score > 6 [*n* = 1]; no measurable lesion [*n* = 1]; ≥ 50% liver occupation [*n* = 1]). Finally, we enrolled a total of 20 eligible patients in the phase 1 dose de-escalation and the phase 2 dose-expansion cohorts (Fig. 1). Patient characteristics are shown in Table 1. The median age of enrolled patients was 60.5 years; of these, 75% were male. 60% of patients had a baseline ECOG performance status of 1. This study included 18 (90%) patients with hepatitis B virus (HBV) infection, and 1(5%) with hepatitis C virus (HCV) infection. Extrahepatic metastases were present in 19 (95%) patients and macrovascular invasion was present in 2 (10%) patients. Patients were heavily pretreated and all of them had previously been treated with systemic therapy, including sorafenib (19 [95%]) and lenvatinib (1 [5%]). During the study, 17 (85%) of 20 patients discontinued treatment; among them, 16 (80%) discontinued due to disease progression or symptomatic deterioration, and one (5%) discontinued due to consent withdrawal. As of data cutoff, 3 patients completed 52-cycle treatment with GT90001 plus nivolumab and the safety follow-up. The last patient's last visit date was 28 April 2022.

**Table 1:** Patient characteristics at baseline

Characteristic	All patients (*n* = 20)
Median age, years	60.5
≤ 65	13 (65.0)
> 65	7 (35.0)
Gender, n (%)	
Male	15 (75.0)
Female	5 (25.0)
ECOG performance status, n (%)	
0	8 (40.0)
1	12 (60.0)
Previous therapy, n (%)	
Systemic therapy	20 (100.0)
Sorafenib	19 (95.0%)
Lenvatinib	1 (5.0%)
Surgical resection	14 (70.0)
Radiotherapy	6 (30.0)
Other treatment	16 (80.0)
By Etiology, n (%)	
HBV	18 (90.0)
HCV	1 (5.0)
α-fetoprotein, n (%)	
≤ 200 ng/mL	9 (45.0)
> 200 ng/mL	11 (55.0)
Extrahepatic metastases, n (%)	19 (95.0)
Macrovascular invasion, n (%)	2 (10.0)
BCLC stage, n (%)	
B	2 (10.0)
C	18 (90.0)
No alcohol dependency or abuse within 1 year, n (%)	20 (100.0)

Data are median or n (%)

*ECOG* Eastern Cooperative Oncology Group, *HBV* hepatitis B virus, *BCLC* Barcelona Clinic Liver Cancer

### DLTs

In the dose de-escalation phase, only 1 patient of the first 6 patients required dose interruption between cycles 1 and 2, because of sepsis (considered unlikely related to the study drug) and renal impairment (considered possibly related to the study drug). One patient reported grade 3 platelet count decreased but had no active bleeding. Other 4 patients experienced grade 2 or less TEAEs within the first 28 days of treatment. In general, GT90001 7.0 mg/kg was well tolerated and no DLTs were observed in the 7.0 mg/kg cohort. Therefore, no dose de-escalation was required and GT90001 7.0 mg/kg was confirmed as the RP2D when combined with nivolumab.

### Safety and tolerability

All 20 patients treated with GT90001 plus nivolumab were assessed for safety. A summary of TEAEs in all grades and grades 3 or 4 in the safety population was shown in Table 2. All patients (100%) experienced TEAEs (all causality); of these, 15 (75%) patients experienced protocol-defined AESI. The most common frequent TEAEs (all causality) in more than 20% of patients were platelet count decreased (11 [55%] of 20 patients, of whom 3 [15%] had grade ≥ 3), pruritus (9 [45%] of 20 patients, of whom one had grade ≥ 3), rash (8 [40%], two had grade ≥ 3), peripheral edema (7 [35%]; one had grade ≥ 3), abdominal distension (5 [25%]), ALT/AST increased (5 [25%]; one had grade ≥ 3), fatigue (5 [25%]), epistaxis (5 [25%]), cough (4 [20%]), constipation (4 [20%]), and dizziness (4 [20%]). A total of 11 (55%) patients occurred grade 3 or 4 AEs, with the most common being platelet count decreased (15%). Grade 3 or 4 AEs in 6 (30%) patients were considered to be treatment-related and included skin rash (*n* = 2), platelet count decreased (*n* = 3), and AST increased (*n* = 1). All these treatment-related grade 3 or 4 events were resolved with supportive care.

**Table 2:** Adverse events in the safety population (*n* = 20)

Adverse events	Adverse events (All-causality) ^a^		Treatment-related^c^	
	All Grades	Grade 3/4	All Grades	Grade 3/4
Any events	20 (100)	11 (55)	20 (100)	6 (30)
Any serious events ^b^	5 (25)	3 (15)	3 (15)	0
Frequent AEs (≥ 10% incidence)				
Platelet count decreased	11 (55)	3 (15)	11 (55)	3 (15)
Pruritus	9 (45)	1 (5)	8 (40)	1 (5)
Rash	8 (40)	2 (10)	8 (40)	2 (10)
Peripheral edema	7 (35)	1 (5)	2 (10)	0
Abdominal distension	5 (25)	0	0	0
ALT/AST increased	5 (25)	1 (5)	5 (25)	1 (5)
Fatigue	5 (25)	0	2 (10)	0
Epistaxis	5 (25)	0	4 (20)	0
Dizziness	4 (20)	0	0	0
Constipation	4 (20)	0	0	0
Cough	4 (20)	0	0	0
Diarrhoea	3 (15)	0	1 (5)	0
Blood bilirubin increased	3 (15)	0	2 (10)	0
Hot flush	3 (15)	0	2 (10)	0
Hypertension	3 (15)	0	3 (15)	0
Decreased appetite	3 (15)	0	0	0
Hypokalaemia	3 (15)	0	0	0
Headache	3 (15)	0	3 (15)	0
Hyperthyroidism	3 (15)	0	3 (15)	0
Jaundice cholestatic	2 (10)	2 (10)	0	0
Pneumonia	2 (10)	1 (5)	0	0
Stomatitis	2 (10)	0	0	0
Chest pain	2 (10)	0	0	0
Blood thyroid stimulating hormone increased	2 (10)	0	2 (10)	0
Weight increased	2 (10)	0	0	0
Skin infection	2 (10)	0	0	0
Upper respiratory tract infection	2 (10)	0	0	0
Dyspnoea	2 (10)	0	1 (5)	0
Rhinitis allergic	2 (10)	0	1 (5)	0
Nausea	2 (10)	0	0	0
Chronic kidney disease	2 (10)	0	0	0
Haematuria	2 (10)	0	0	0
Abdominal pain	2 (10)	0	0	0
Eosinophilia	2 (10)	0	2 (10)	0
Insomnia	2 (10)	0	0	0
Benign prostatic hyperplasia	2 (10)	0	0	0

Data were expressed as n (%)

^a^ Adverse events were defined as all treatment-emergent adverse events regardless of relationship to study drug

^b^ Serious events were defined as events that result in death, hospital admission or prolongation of a hospital admission, persistent or significant disability/incapability, congenital anomaly/birth defect, or were life-threatening, or any other medically important events

^c^ Treatment-related events were identified by the investigator and defined as events that are “possibly”, “probably”, or “definitely” related to GT90001, nivolumab, or both

A total of 7 SAEs were reported in five (25%) patients, and the events included hepatitis [*n* = 1], jaundice cholestatic [*n* = 1], hyperamylasemia [*n* = 1], hyponatraemia [*n* = 1], sepsis [*n* = 1], renal impairment [*n* = 1], and metastases to central nervous system [*n* = 1]. Among them, hepatitis, hyperamylasemia, and renal impairment were judged to be treatment-related (15%). During the whole study, only 2 (10%) patients required dose reduction due to grade 3 platelet count decreased, which were considered to be probably related to GT90001 (Appendix File 1: Table S2). Thus, the dose of GT90001 was reduced from 7.0 to 4.5 mg/kg and the dose of nivolumab was withheld. Additionally, nivolumab was withheld in 1 patient due to skin rash and another 1 due to ALT/AST increased and nephropathy. GT90001 was withheld in 1 patient due to platelet count decreased. Treatment interruption of both GT90001 and nivolumab due to TEAEs occurred in 9 (60%) patients (Appendix File 1: Table S2). The most common cause was platelet count decreased (2 patients). In all other cases, supportive care was sufficient to decrease the severity to grade 1 or less. No TEAE-related treatment discontinuation or deaths occurred.

Although most patients experienced some increase in systolic and diastolic blood pressure (Appendix File 1: Figure S2), only 3 of them were recorded as having hypertension as an adverse event according to NCI-CTCAE 5.0 (Table 2). No evident association between blood pressure changes and response to study drug treatment was noted (data not shown).

A total of 6 patients occurred irAEs that required steroid therapy. Among them, patient 1 (Pt1) experienced skin rash and recovered from topical desoximetasone ointment (twice/day, 4 weeks) and oral prednisolone (15–30 mg/day, 2 weeks). However, Pt2’s skin rash remained even after 7-week topical desoximetasone and 1-year oral prednisolone (5–30 mg/day). In addition, one patient reported two suspected pneumonitis events and he recovered after oral dexamethasone (4 mg, 7 days) followed by prednisolone (2–30 mg/day, 1 month). Another 3 patients received dexamethasone therapy to manage diabetes insipindus (subcutaneous injection, 4 mg), brain metastasis post-treatment sequela (IV, 5 mg), and oral ulcer (topical, 2 weeks as needed), and all these events were resolved.

### Efficacy

**Fig. 2:**
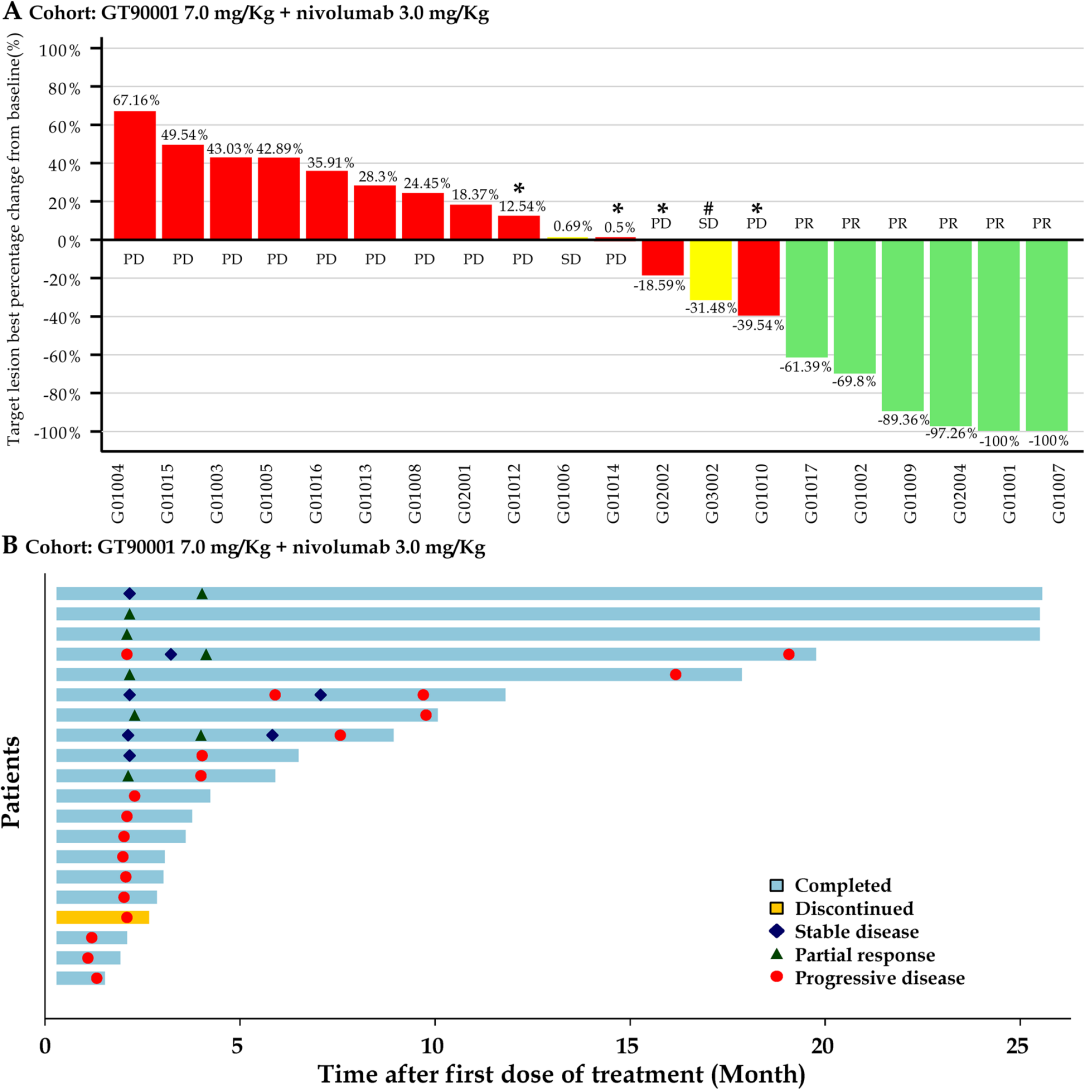
Tumor response. **A** Waterfall plot of maximum percent change in tumor size from baseline in each patient as measured by Response Evaluation Criteria in Solid Tumors (version 1.1). *: patients whose PD status was documented because of appearance of new lesions. #: patients whose SD status documented because of unconfirmed PR. **B** Time to response and duration of response. Each bar represents one patient

**Fig. 3:**
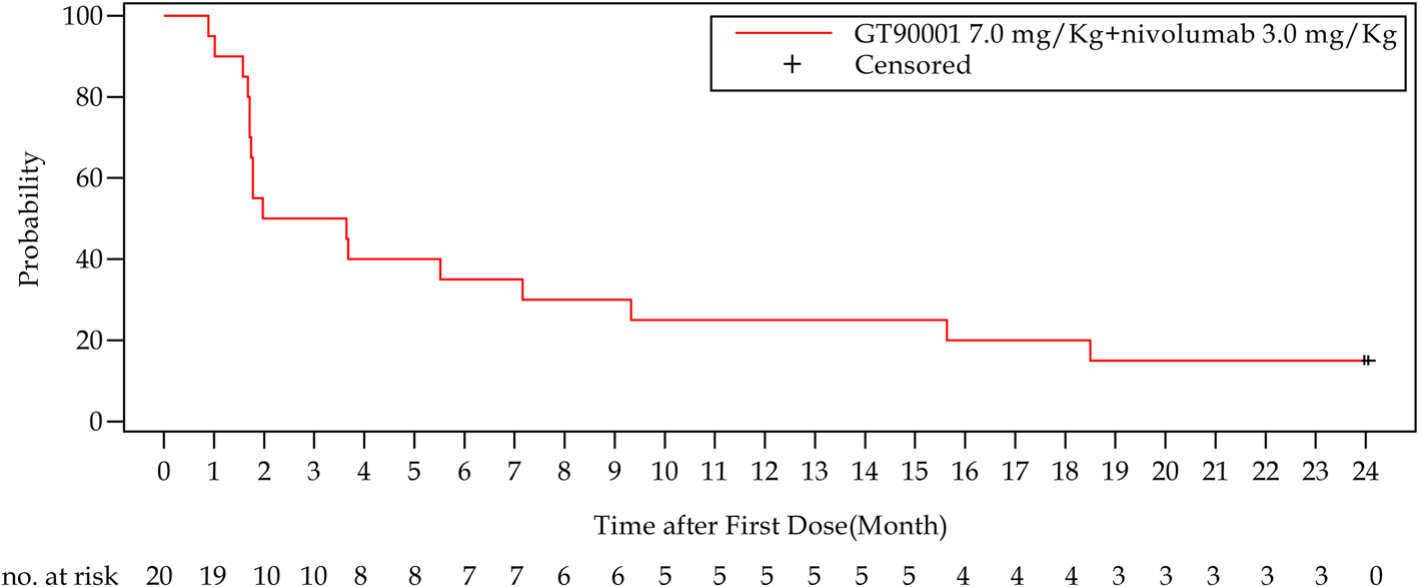
Kaplan–Meier curves of progression-free survival

In phases 1b and 2, a total of 20 patients were enrolled in the GT90001 7.0 mg/kg dose level and evaluable for response. Target lesion size decreased in 9 (45%) patients by investigator assessment (Fig. 2A). Objective responses were observed in 8 (40%; 95% CI, 19.1% to 63.9%) of 20 patients by investigator assessment; of these eight patients, all patients achieved PR. The reassessment was performed for patients at week 4 from PR or CR being reported for the first time, as part of a work-up for the response confirmation. Six (30%) patients achieved a confirmed PR (Table 3), with a confirmed ORR of 30% (95% CI, 14.6% to 51.9%). Of the 6 responders, 5 (83.3%) patients had a DOR of more than 6 months, and 4 (66.7%) patients had a DOR of more than 12 months. The median DOR was not reached (NR; 95% CI, 7.39 months to NR) and the median TTR was 1.91 months (95% CI, 1.17 to NR). The time on treatment for all patients is shown in Fig. 2B. SD (≥ 16 weeks) was reported as the best response in 2 (10%) patients. Overall, the confirmed DCR with SD ≥ 16 weeks was 40% (95% CI, 21.9% to 61.3%). The number of PFS events was 17 at the data cutoff and median PFS was 2.81 months (95% CI, 1.71 to 9.33 months). The 6-month and 12-month PFS were 35% (95% CI, 15.7% to 55.2%) and 25% (95% CI, 9.1% to 44.9%), respectively (Fig. 3).

**Table 3:** Activity of GT90001 plus nivolumab

	Patients (*n* = 20)
Confirmed objective response^a^	6 (30%; 14.6%-51.9%)
Partial response	6 (30%; 14.6%-51.9%)
Stable disease (≥ 16 weeks)	2 (10%; 2.8%-30.1%)
Progressive disease	12 (60%; 38.7%-78.2)
Confirmed disease control^b^	8 (40%; 21.9%-61.3%)
Median time to response	1.91 months (1.17-NC)
Median duration of response	NC (7.39-NC)
Median progression-free survival	2.81 months (1.71–9.33)

Data are expressed as n (%; 95% CI) or median (95% CI)

*CI* confidence interval, *NC* not calculated

^a^ Objective response = complete response or partial response

^b^ Disease control = complete response, partial response, or stable disease

**Fig. 4:**
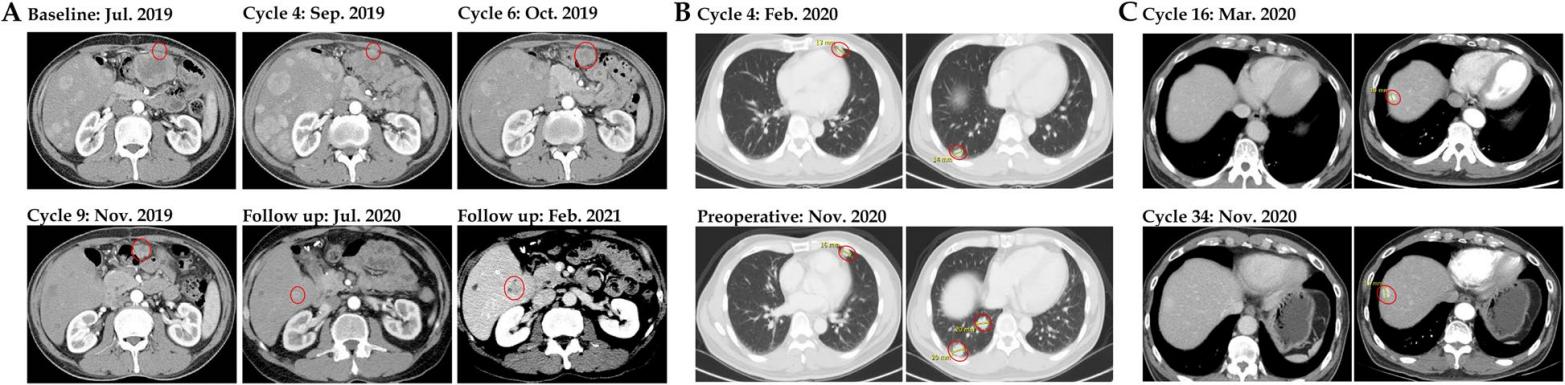
CT images of representative cases. **A** Patient 2 experienced pseudo-progression after 4 cycles of GT90001 plus nivolumab treatment and achieved partial response at cycle 6. **B** Patient 6 with lung metastases achieved stable disease under treatment while also experiencing progressive enlargement of lung lesions in February 2020. After surgical resection for lung lesions in November 2020, the patient remained recurrence-free for more than 6 months. **C** Patient 1 with lung metastases achieved PR under the treatment but experienced HCC recurrence

One patient (Pt2, 54-year-old man) was diagnosed with pseudo-progression upon GT90001 plus nivolumab exposure (Fig. 4A). This patient with multiple intra- and extra-hepatic metastases started study treatment on August 5, 2019. A CT scan showed enlargement of the liver lesions after two-cycle treatment (September 2, 2019). Laboratory tests confirmed the increase of alpha-fetoprotein (AFP) and liver function indexes (AST and bilirubin) at the time of pseudo-progression. Due to the improvement of symptoms, the patient received post-progression therapy. From week 10 (October 14, 2019), the liver lesions looked necrotic and the abdominal tumor continued to shrink, accompanied by a decrease of AFP and AST levels. At the one-year follow-up examination (July 2020), the CT scan revealed a high-density lesion, raising the suspicion of tumor progression. In March 2021, the patient presented with progression in CT showing enlargement of liver lesions. Thus, a biopsy was collected for diagnostic purposes and HCC recurrence was proven. The patient underwent ablation therapy. Since then, the patient had no evidence of progression and remained recurrence-free for more than 6 months. During the treatment, he experienced a skin rash after GT90001 plus nivolumab exposure. To manage the toxicity, he received topical dexametasone ointment twice/day for 7 weeks, followed by 5–30 mg/day prednisolone (from February 4, 2020 to February 3, 2021), but the skin rash fluctuated until the end of follow-up. Another patient (Pt6, 52-year-old man) with lung metastases started study treatment on December 2019 and has achieved the best response of SD. However, the follow-up CT images showed progressive enlargement of the lung lesions from February 2020 (Fig. 4B). Then, the patient underwent surgical resection for lung metastases in November 2020 and remained recurrence-free for more than 6 months. Pt1 (60-year-old man) with lung metastases started a study treatment since July 2019 and achieved the best response of PR. The CT scan showed the possible recurrence of HCC (lesion increase from 10 to 17 mm) (Fig. 4C). The suspicion of HCC recurrence was disproved by the surgery. Furthermore, the combination of GT90001 and nivolumab shows a remarkable long-tail effect, since 8 subjects are still alive as of 19^th^ Apr 2023. 6 of them have received no further systematic anti-tumor therapy after trial completion. And 3 patients are still on progression-free status without any other anti-tumor treatment.

## Discussion

On the basis of evidence showing the potential of antiangiogenic agents plus immunotherapy, we conducted the first, multicenter, single-arm, phase 1b/2 study to determine the recommended phase 2 dose of GT90001 when combined with nivolumab, as well as to prospectively investigate the efficacy and safety of this combination at the recommended dose in patients with advanced HCC. In this phase 1b/2 study, we found that this combination regimen is well-tolerated and has promising anti-tumor activity with durable remissions and objective responses in this population.

The safety profiles of GT90001 plus nivolumab are generally acceptable and manageable in our patients. The most common events were platelet count decreased, fatigue, epistaxis, dizziness, and constipation but these events of grade 3 or more were infrequent, which was consistent with the historical data for solid tumors [16]. More importantly, these events were resolved soon with supportive care or dose interruptions. Only 9 patients required dose interruptions due to TEAEs, and 6 patients received steroid therapy to manage skin rash, suspected pneumonitis, brain metastasis post-treatment sequela, and oral ulcer. In all other cases, supportive care was sufficient to decrease the severity to grade 1 or less. No TEAEs leading to dose reduction, discontinuation, or deaths occurred. The most-reported irAEs were rash, peripheral edema, and diarrhea, while these events were as expected, mild, infrequent, and manageable. The safety profiles of this regimen were consistent with the known profiles of GT90001 or nivolumab monotherapies in similar populations or other tumor types [2, 18, 20, 21]. Telangiectasia is a major safety concern for ALK-1-targeted agents but was not observed in our study. Overall, no new and unexpected safety signals were identified for the GT90001 plus nivolumab regimen, suggesting that they can be delivered safely with acceptable toxicity in this population.

Anti-PD1/anti-PDL1 plus anti-angiogenic agents or anti-CTLA4 regimens are now the standard of care as first-line systemic therapy for unresectable HCC based on the survival benefit, compared with single-agent multi-kinase inhibitor (MKI) therapy, demonstrated by randomized controlled trials [22–24]. Although these clinical trials greatly improve our care for HCC patients, several scientific and clinical questions remain unanswered. First, no clinical characteristics or predictive biomarkers can be used to select one regimen over another based on the trial results. Second, in the case of anti-angiogenic MKI, not all trials were positive. The mechanistic reasons for the lack of improved survival compared with MKI monotherapy (e.g., cabozantinib plus atezolizumab versus sorafenib [25], pembrolizumab plus lenvatinib versus lenvatinib [26]) are unknown. Third, treatment options for patients whose tumors progress after first-line anti-PD1/anti-PDL1 based therapy are lacking, and the development scope of second-line systemic therapy is still elusive. Agents with novel immune modulatory mechanisms are needed.

GT90001 may modulate the tumor immune microenvironment through its TGF-β-inhibiting and anti-vascular effects. While single-agent GT90001 therapy had only limited antitumor efficacy in advanced solid tumors (ORR of 0–6.8%, median PFS shorter than 2 months [16–18]), combination of GT90001 with nivolumab in this trial demonstrated promising response rate and long-term tumor control in the responding subjects. In 2 of our subjects, even when new recurrent tumors developed after study drug therapy, post-progression surgery or ablation therapy achieved recurrence-free status for more than 6 months and the original wide-spread metastases remained in remission. This finding suggests that the combination of GT90001 and nivolumab might induce long-term immune-modulatory effects. A biomarker study of GT90001 monotherapy in patients with advanced HCC suggested that the following markers were associated with treatment efficacy: high tumor expression of c-met, high serum levels of bone morphogenetic protein-9 (a high affinity ALK-1 ligand), lower serum TGF-β, and low vascular endothelial growth factor receptor-3 [18]. These findings imply the pleotropic immune modulatory effects of GT90001 through modulation of both TGF-β and angiogenesis signaling.

An obvious limitation of this study is its small sample size and non-randomized design, which make comparison of treatment efficacy of GT90001 plus nivolumab with other ICI or MKI-based regimens difficult. This study did not prospectively collect tumor or blood samples for biomarker studies, which is critical for mechanistic exploration. Finally, all of our subjects received first-line MKI therapy (sorafenib or lenvatinib). Application of results from this study will be limited because the standard first-line systemic therapy for unresectable HCC now is ICI-based combination therapy. This change will also impact on design of confirmatory clinical trials of new regimens in the second-line setting. Despite all these limitations, future studies for mechanistic clarification and structure optimization of ALK-1 inhibitor are warranted to identify novel immune modulatory agents for the treatment of HCC.

## Conclusions

This phase 1b/2 study confirmed that the combination of GT90001 (7.0 mg/kg, every 2 weeks) and nivolumab had a manageable safety profile in patients with advanced HCC. Additionally, the regimen demonstrated promising anti-tumor activity, showing durable remissions and objective responses in this population. This finding suggested that this combination might be a potential treatment option for advanced HCC.

### Supplementary Information

**Additional file 1:** **Appendix Table 1.** Complete eligibility criteria. **Appendix Table S2.** Details of dose reduction or interruption due to treatment-emergent adverse events in all patients. **Appendix Figure S1.** Study procedures. **Appendix Figure S2.** Changes in patient blood pressure. (A) Changes in blood pressure between baseline and at the end of study drug treatment. (B) The percentage of patients who experienced a change in blood pressure. Abbreviations: SBP=systolic blood pressure; DBP=diastolic blood pressure.

## Data Availability

The datasets supporting the results of the present study can be obtained from the corresponding author upon reasonable request.

## Abbreviations

AE: Adverse event
AFP: Alpha fetoprotein
ALK-1: Activin receptor-like kinase 1
ALT: Alanine aminotransferase
AST: Aspartate aminotransferase
CIs: Confidence intervals
CR: Complete response
CT: Computed tomography
DCR: Disease control rate
DLTs: Dose-limiting toxicities
DOR: Duration of response
ECOG: Eastern Cooperative Oncology Group
HBV: Hepatitis B virus
HCC: Hepatocellular carcinoma
HCV: Hepatitis C virus
ICH-GCP: International Council for Harmonisation Good Clinical Practice
ICIs: Immune checkpoint inhibitors
IO: Immuno-oncology
IQR: Interquartile range
irAEs: Immune-related adverse events
IV: Intravenous infusion
MKI: Multi-kinase inhibitor
NCI-CTCAE: National Cancer Institute Common Terminology Criteria for Adverse Events
NR: Not reached
ORR: Objective response rate
PD: Disease progression
PD1: Programmed cell death-1
PDL1: Programmed cell death ligand-1
PFS: Progression-free survival
PR: Partial response
RP2D: Recommended phase 2 dosage
SAEs: Serious adverse events
SD: Stable disease
TEAEs: Treatment-emergent adverse events
TGFβ: Transforming growth factor-β
TTR: Time to response
VEGFR: Vascular endothelial growth factor receptor
