# Supplementary material for: Combination of GT90001 and nivolumab in patients with advanced hepatocellular carcinoma: a multicenter, single-arm, phase 1b/2 study
Source: BMC Med. 2023 Oct 20;21:395. doi: 10.1186/s12916-023-03098-w (PMC10588186; doi:10.1186/s12916-023-03098-w)

# Additional file 1

# Table S1. Complete eligibility criteria.

| **Inclusion criteria** |
| --- |
| All subjects participating in this clinical trial must meet all of the following criteria:  1. Be willing and able to provide written informed consent for the trial;  2. Age ≥20 years male and female;  3. Subjects must have confirmed diagnosis of unresectable HCC with any of following criteria:  i. Histologically or cytologically confirmed diagnosis of HCC  ii. Have Barcelona Clinic Liver Cancer (BCLC) Stage C disease or BCLC Stage B disease not amenable to locoregional therapy or refractory to locoregional therapy;  4. Have documented disease progression or intolerance after first-line systemic treatment;  5. At least one measurable lesion based on RECIST version 1.1;  6. Child-Pugh score ≤6 (Child-Pugh A) score within 7 days of first dose of study drug;  7. ECOG performance status: 0-1;  8. Have a predicted life expectancy of greater than 3 months;  9. The functions of the important organs are confirmed with the following requirement:  • Hemoglobin (HGB) ≥90 g/L;  • White blood cell count (WBC) ≥3×10^9^/L；  • Absolute neutrophil count (ANC) ≥1.5×10^9^/L；  • Platelets (PLT) ≥75×10^9^/L；  • Total bilirubin (TBIL) ≤ 1.5× Upper limit of normal value (ULN)  • Aspartate aminotransferase (AST), alkaline phosphatase (ALP), and alanine aminotransferase (ALT) ≤5× ULN  • Creatinine (Cr) ≤1.5×ULN；  • International normalization ratio (INR)or prothrombin time (PT) ≤1.5×ULN ;  10. Women must have a negative serum or urine pregnancy test within 72 hours prior to the start of investigational product;  11. Women of childbearing potential must agree to contraception for the duration of study treatment and 5 months after the last dose of study treatment;  12. Willing and able to comply with all aspects of the protocol |
| **Exclusion criteria** |
| An individual who meets any of the following criteria will be excluded from participation in this study:  1. Imaging findings for HCC corresponding to any of the following:  • HCC with ≥50% liver occupation  • Clear invasion into the bile duct  • Portal vein invasion or thrombosis at the main portal branch (Vp4)  2. Gastric or esophageal varices that require treatment;  3. If prior history of DVT/PE, the patient needs to be on stable doses of anticoagulation with low molecular weight heparin or oral anticoagulant for at least two weeks;  4. Esophageal vein dilation, grade A of active peptic ulcer rating, and all bleeding risk by gastroscopy;  5. History of arterial thromboembolic event in past 6 months;  6. Active bleeding disorder, including gastrointestinal bleeding event or active hemoptysis within 28 days prior to study treatment;  7. Have central nervous system (CNS) metastases;  8. Has a known history of human immunodeficiency virus (HIV);  9. Has received prior immune checkpoint inhibitor (including those targeting PD-1, PD-L1 or PD-L2, CD137, or cytotoxic T-lymphocyte antigen [CTLA-4]);  10. Has a known history of, or any evidence of, interstitial lung disease or active non- infectious pneumonitis;  11. Has active autoimmune disease that has required systemic treatment in past 2 years;  12. Has known psychiatric or substance abuse disorders that would interfere with cooperation with the requirements of the trial;  13. Any history of drug or alcohol dependency or abuse within the prior 1 years;  14. Has known active Hepatitis B or Hepatitis C within 2 weeks prior to initiation of study treatment; Note: Patients with HBV infection are required to be receiving effective antiviral therapy over two weeks, and then have continuous therapy in study period;  15. Pregnant, breast feeding, or planning to become pregnant;  16. Have a history of severe hypersensitivity reaction to monoclonal antibody;  17. Significant cardiovascular impairment: history of congestive heart failure greater than New York Heart Association (NYHA) Class II, unstable angina, myocardial infarction or stroke within 6 months of the first dose of study drug;  18. Have surgery, radiotherapy, ablation within one month before screening;  19. Subjects with any other serious disease considered by the investigator not in the condition to enter into the trial |

# Table S2. Details of dose reduction or interruption due to treatment-emergent adverse events in all patients.

|  | **Subject ID** | **Event Name** | **Onset Date** | **Date Resolved** | **AE grade** | **Relationship to Nivolumb** | **Relationship to GT90001** | **Action Taken with GT90001** | **Action Taken with Nivolumb** | **Outcome** |
| --- | --- | --- | --- | --- | --- | --- | --- | --- | --- | --- |
| Dose de-escalation phase | G01002 | Rash | 2020/01/14 | 2020/02/04 | Grade 2 | Possibly related | Unlikely to be related | Dose not changed | Drug withhold | Not recovered/Not resolved |
|  |  | Rash | 2020/02/04 | 2020/02/17 | Grade 3 | Possibly related | Unlikely to be related | Dose not changed | Drug withhold | Recovering/Resolving |
|  |  | Rash | 2020/02/18 | 2020/03/03 | Grade 2 | Possibly related | Unlikely to be related | Dose not changed | Drug withhold | Recovering/Resolving |
|  |  | Rash | 2020/03/17 | 2020/03/30 | Grade 2 | Possibly related | Possibly related | Dose not changed | Drug withhold | Recovering/Resolving |
|  |  | Rash | 2020/10/22 | 2020/11/26 | Grade 3 | Possibly related | Possibly related | Dose not changed | Drug withhold | Recovering/Resolving |
|  |  | Rash | 2020/11/26 | 2020/12/10 | Grade 2 | Possibly related | Possibly related | Dose not changed | Drug withhold | Recovering/Resolving |
|  | G01003 | Platelet count decreased | 2019/08/19 | 2019/09/02 | Grade 3 | Unlikely to be related | Possibly related | Drug withhold | Drug withhold | Recovering/Resolving |
|  |  | Platelet count decreased | 2019/09/03 | 2019/09/03 | Grade 2 | Unlikely to be related | Possibly related | Drug withhold | Drug withhold | Not recovered/Not resolved |
|  |  | Platelet count decreased | 2019/09/04 | 2019/09/12 | Grade 3 | Unlikely to be related | Possibly related | Drug withhold | Drug withhold | Recovering/Resolving |
|  | G02001 | Renal dysfunction irAE | 2019/8/19 | 2019/8/29 | Grade 2 | Possibly related | Possibly related | Drug withhold | Drug withhold | Recovering/Resolving |
|  |  | Renal dysfunction irAE | 2019/8/29 | 2019/10/3 | Grade 1 | Possibly related | Possibly related | Drug withhold | Drug withhold | Recovered/Resolved |
|  |  | Sepsis | 2019/8/31 | 2019/9/2 | Grade 3 | Unlikely to be related | Unlikely to be related | Drug withhold | Drug withhold | Recovered/Resolved |
| Dose-expansion phase | G01006 | Suspected immue-related nephropathy | 2020/05/11 | 2020/05/25 | Grade 1 | Possibly related | Unlikely to be related | Drug withhold | Drug withhold | Recovered/Resolved |
|  | G01008 | Suspected immune-related hepatotoxicity | 2020/02/25 | 2020/04/06 | Grade 2 | Probably related | Unlikely to be related | Drug withhold | Drug withhold | Recovered/Resolved |
|  | G01012 | Platelet count decreased | 2020/04/27 | 2020/05/04 | Grade 3 | Unlikely to be related | Probably related | Drug withhold | Drug withhold | Recovering/Resolving |
|  |  | Platelet count decreased | 2020/05/04 | 2020/05/25 | Grade 2 | Unlikely to be related | Probably related | Dose Reduced | Dose not changed | Not recovered/Not resolved |
|  | G01014 | Obstructive janudice | 2020/06/22 |  | Grade 3 | Not related | Not related | Drug withhold | Drug withhold | Not recovered/Not resolved |
|  | G01017 | Hyponatremia | 2021/07/21 | 2021/07/23 | Grade 3 | Not related | Not related | Drug withhold | Drug withhold | Recovered/Resolved |
|  |  | Platelet count decreased | 2020/06/22 | 2020/07/20 | Grade 3 | Unlikely to be related | Probably related | Drug withhold | Dose not changed | Recovering/Resolving |
|  |  | Platelet count decreased | 2020/07/20 | 2020/08/18 | Grade 2 | Unlikely to be related | Probably related | Dose Reduced | Dose not changed | Not recovered/Not resolved |
|  |  | Platelet count decreased | 2020/08/18 | 2020/09/15 | Grade 3 | Unlikely to be related | Probably related | Drug withhold | Dose not changed | Recovering/Resolving |
|  |  | Platelet count decreased | 2020/09/15 | 2020/10/14 | Grade 2 | Unlikely to be related | Probably related | Dose Reduced | Dose not changed | Not recovered/Not resolved |
|  |  | Platelet count decreased | 2020/10/14 | 2020/11/10 | Grade 3 | Unlikely to be related | Probably related | Drug withhold | Dose not changed | Recovering/Resolving |
|  | G02004 | Hyperamylasemia | 2020/5/8 | 2020/5/17 | Grade 2 | Possibly related | Unlikely to be related | Drug withhold | Drug withhold | Recovering/Resolving |
|  | G03002 | Aspartate aminotransferase increased | 2020/08/06 | 2020/08/11 | Grade 3 | Probably related | Probably related | Drug withhold | Drug withhold | Recovered/Resolved |

# Figure S1. Study procedures.


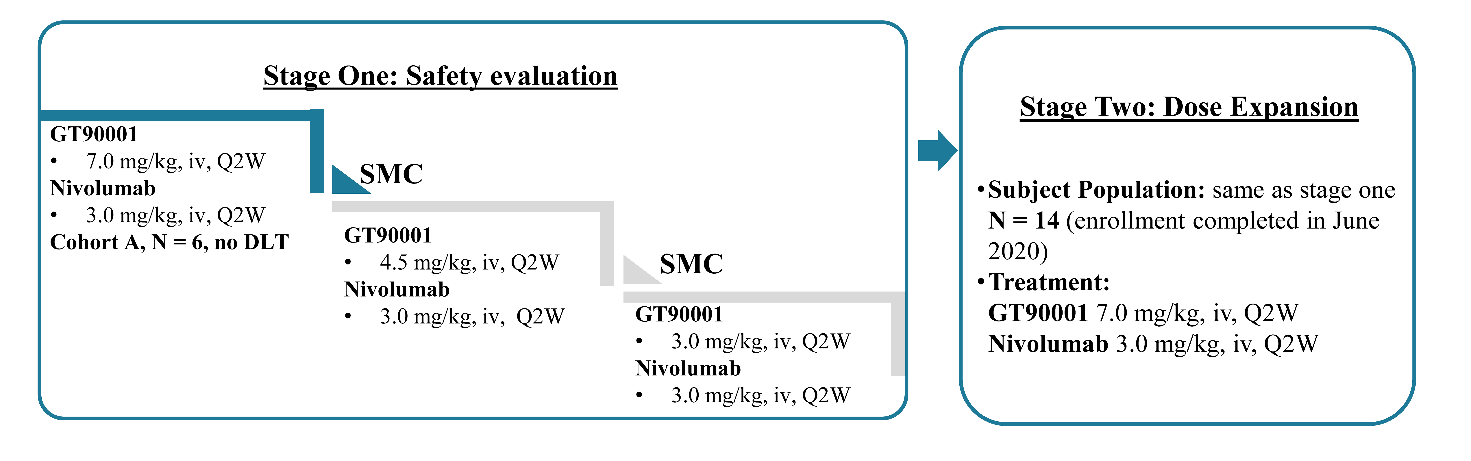


# Figure S2. Changes in patient blood pressure.

(A) Changes in blood pressure between baseline and at the end of study drug treatment. (B) The percentage of patients who experienced a change in blood pressure. Abbreviations: SBP=systolic blood pressure; DBP=diastolic blood pressure.


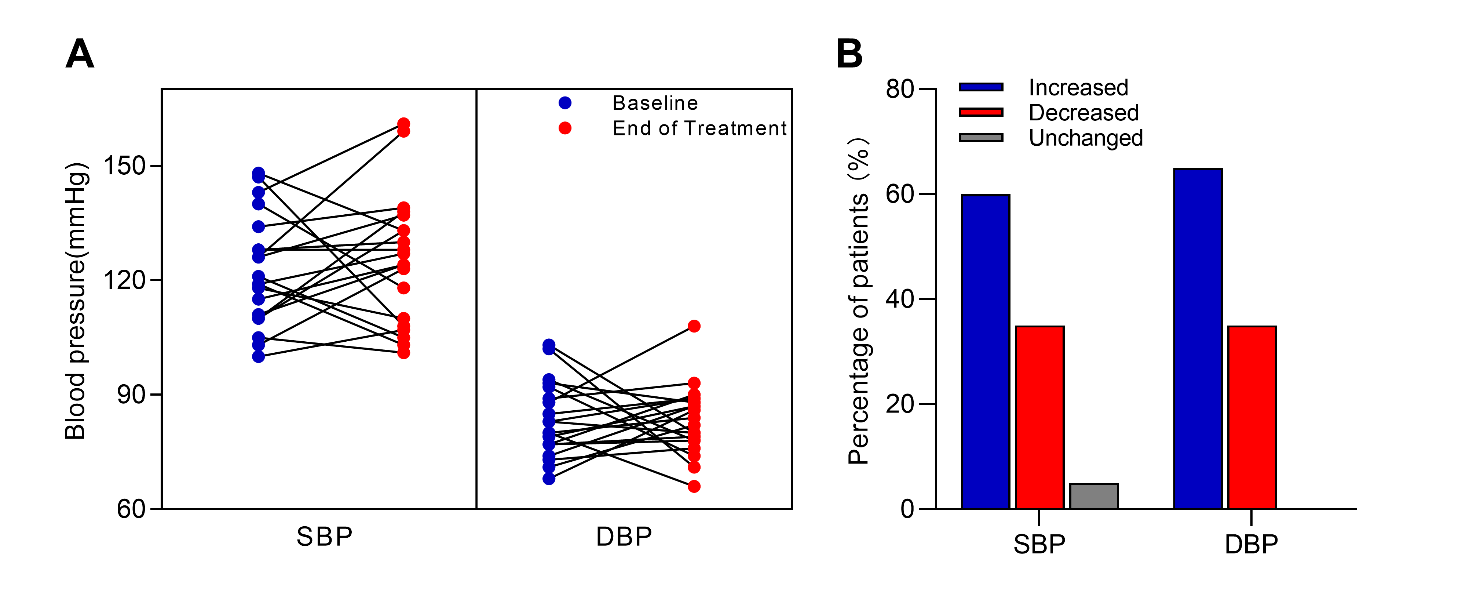

Supplement: Supplementary file 1 — Additional file 1: Appendix Table 1. Complete eligibility criteria. Appendix Table S2. Details of dose reduction or interruption due to treatment-emergent adverse events in all patients. Appendix Figure S1. Study procedures. Appendix Figure S2. Changes in patient blood pressure. (A) Changes in blood pressure between baseline and at the end of study drug treatment. (B) The percentage of patients who experienced a change in blood pressure. Abbreviations: SBP=systolic blood pressure; DBP=diastolic blood pressure. [file 12916_2023_3098_MOESM1_ESM.docx]
